# The Possible Role of Epigenetics in Gestational Diabetes: Cause, Consequence, or Both

**DOI:** 10.1155/2010/605163

**Published:** 2010-10-31

**Authors:** J. L. Fernández-Morera, S. Rodríguez-Rodero, E. Menéndez-Torre, M. F. Fraga

**Affiliations:** ^1^Endocrinology and Nutrition Service, Hospital Universitario Central de Asturias, Av. Julian Clavería s/n, 33006 Oviedo, Spain; ^2^Cancer Epigenetics Laboratory, Instituto Universitario de Oncología del Principado de Asturias (IUOPA), Universidad de Oviedo, HUCA, 33006 Oviedo, Spain; ^3^Department of Immunology and Oncology, National Center for Biotechnology, CNB-CSIC, Cantoblanco, 28049 Madrid, Spain

## Abstract

Gestational diabetes mellitus (GDM) is defined as the glucose intolerance that is not present or recognized prior to pregnancy. Several risk factors of GDM depend on environmental factors that are thought to regulate the genome through epigenetic mechanisms. Thus, epigenetic regulation could be involved in the development of GDM. In addition, the adverse intrauterine environment in patients with GDM could also have a negative impact on the establishment of the epigenomes of the offspring.

## 1. Gestational Diabetes Mellitus

Late pregnancy is characterized by moderate peripheral insulin resistance and hyperinsulinemia, both of which are necessary to ensure an appropriate supply of nutrient to the fetus. It is counteracted by the adaptation of the islets of Langerhans to the higher insulin demand. This adaptation is characterized by an increase of insulin biosynthesis, enhanced glucose-stimulated insulin secretion, and an increase of b-cell mass. It is not completely understood why, in some individuals, the b-cell mass and function fail to adapt to the metabolic demands of pregnancy and why blood glucose concentration rises to pathological levels [1, 2]. Gestational diabetes mellitus (GDM) is defined as glucose intolerance that was not present or recognized prior to pregnancy and it is diagnosed when the pancreatic function in women is not sufficient to control the diabetogenic environment that pregnancy confers [3]. The diagnosis of GDM also identifies pregnancies at increased risk of perinatal morbidity [4]. 

The incidence of GDM differs among ethnic populations, with higher rates in African American, Hispanic, American Indian, and Asian women than in white women; values range from 1.4% to 14% but overall the condition commonly affects between 2% and 5% of pregnant women [5]. 

The frequency of GDM varies in direct proportion to the prevalence of type II diabetes in populations, and women who develop GDM during pregnancy have a higher risk of developing type 2 diabetes (T2D) later in their lives. These observations are important for relating the two pathological situations and they probably arise from common physiopathological mechanisms [6]. 

There are two different methods for classifying diabetes in pregnancy. The most recent is the American Diabetes Association (ADA) classification [7] but the White classification is still useful [8] even though it was published in 1949.

### 1.1. Risk Factors for GDM

Several risk factors are associated with the development of GDM. The most common is obesity (body mass index over 30) diagnosed before pregnancy [9]. Being a member of an ethnic group with a higher rate of type II diabetes (as mentioned above), polycystic ovarian syndrome [10], essential hypertension, or pregnancy-related hypertension [11], strong family history of diabetes in first-degree relatives and a history of GDM in a previous pregnancy are other important risk factors [12]. Nevertheless, no risk factors are known in around 50% of patients with GDM. 

There is enough evidence to assert that T2D has a strong genetic component. The concordance of T2D in monozygotic twins is approximately 70%, compared with 20%–30% in dizygotic twins [13, 14].

Family studies have prompted the emergence of new perspectives regarding the identification of GDM susceptibility genes. Familial clustering of GDM and T2D have been studied, linking T2D and impaired glucose tolerance in families with GDM [15] and there is evidence of a higher incidence of T2D in mothers of women with GDM [16]. 

The latest genome-wide studies have identified a number of genetic variants that can explain some of the interindividual discrepancies in diabetes susceptibility [17, 18] and different genes have been associated with GDM and T2D [19]. Although classical MHC class II molecules HLA-DR3 and DR4 were not found to be related to GDM [20], recent studies have found genetic evidence from PPARG, KCNJ11, TCF2/HNF1B, and WFS1 or HNF4A that is related to dysfunctional pancreatic b-cell secretion and GDM, suggesting new possibilities for the study of GDM [21, 22].

Some strands of scientific opinion suggest a possible role for epigenetic factors in the complex interplay between genes and the environment that are related to insulin resistance, T2D, and GDM mellitus [23]. These pathological conditions are associated with strong interactions between genetic susceptibility (usually a polygenic contribution) and environment influences over time, such as lifestyle and social influences (e.g., overfeeding, sedentarism, and obesity), or fetal surroundings (epidemiological studies suggest that the perinatal environment can predispose human offspring to developing obesity and T2D). These interactions may bring about the activation and/or deactivation of genes by epigenetic mechanisms, enabling adaptation to these various environmental situations [24].

### 1.2. Pathophysiology and Medical Complications

The pathogenesis of the most common type of GDM have been described similar to that of type 2 diabetes (T2D), with both pancreatic b-cell dysfunction and chronic insulin resistance playing crucial roles [25]. Pregnancy as an insulin resistant state may disclose pre-existing deficiency in insulin secretion or insulin sensitivity being present relative b-cell failure [26, 27].

Moreover, women with GDM after delivery often have an increased risk for metabolic syndrome, and they have been shown to express early markers of vascular dysfunction such as increased intimamedia thickness of carotid arteries [28]. Thus, pregnancy is a stress situation that may reveal predisposition to T2D on women that provide early signs for prevention of essential chronic diseases [29]. High maternal adipose deposition [30] and low levels of exercise [31, 32] also contribute to this state of relative insulin resistnce something that classically occurs in T2D. Other releasing factors, such as TNF*α* [33], leptin [34], human placental lactogen, cortisol, which is an insulin antagonist, estrogen, or progesterone have previously been found to affect the glucose-insulin balance [35].

There are outstanding medical complications that have been related to GDM for mother and also offspring. For mothers, GDM development have been related to overweight and obesity prior pregnancy [36] and also have been recognised as a sign of increased risk of developing type 2 diabetes (T2D) during life [37]. There are studies that have confirmed the risk of developing T2D in previously diagnosed GDM approximately 40% being followed up during ten year [38]. There is an increasing incidence of T2D (is highest in the first 5 years after pregnancy) which is frequently related with a substantial increase in BMI in women with GDM [39]. 

Congenital anomalies are not more common in patients with GDM but there is an increased incidence of stillbirth when glucose control is poor [40]. Organogenesis usually may start in the third week after fertilization, and it is almost completed by the 8th or 10th week of gestation so during the time most women are aware they are pregnant, substantial organogenesis has occurred. Maternal insulin does not cross the placental structures, and foetal beta islets are not developed to produce insulin until 12th week of gestation where early hyperglycemias can lead to congenital malformations and miscarriages, something that is two or three times more likely among patients with type 1 and type 2 diabetes than in healthy pregnancies [41].

This situation is also important because periconceptual hyperglycemia is known to be teratogenic and undiagnosed T2D might have a role in pregnancy outcome [42, 43]. Congenital abnormalities (3% incidence in total population) are not found more frequently in the offspring of patients with GDM when it is mostly diagnosed over 20th week of gestation [44] but abnormalities are more common in the offspring of patients with both T1D and T2D [45, 46].

Recent literature defines macrosomia as identical to large for gestational age (LGA), which is size >90th percentile for gestational age [47] but this term is habitually used as birth weight over 4000 grams. When a fetus (over 12th week of gestation) is exposed to high levels of maternal glucose, it responds by secreting high levels of insulin in its circulation to control these hyperglycemias. This is a “double sword” mechanism where insulin that also has growth hormone properties develops a high tendency for foetal macrosomia and an increased rate of delivery complications [48]. Some risk for macrosomic and LGA infants deliveries may be found in the general population but is found higher in GDM, T2D, or postprandial hyperglycemic patients as well as high maternal prepregnancy weight or maternal age over 40 [49, 50]. 

Moreover, postpartum neonatal hypoglycemia may occur when neonates are no longer exposed to high levels of maternal glucose. At delivery, neonates continue producing elevated rates of insulin, but without high levels of maternal glucose that leads to a virtual neonatal hyperinsulinemia and subsequent hypoglycemias, requiring glucose infusion after delivery [51]. 

Finally, other important complications have been described in offspring of diabetic gestations with repercussions in future. Children who have been diagnosed LGA at birth and were exposed to an intrauterine environment of either diabetes or maternal obesity developed an increased risk of metabolic syndrome (MS) during childhood. This increased rate of obesity prevalence among children and adults may have implications for perpetuating the cycle of obesity, insulin resistance, and their consequences (GDM, T2DM, MS, and cardiovascular diseases) in subsequent generations [52–54].

## 2. Epigenetic Regulation

Epigenetics is currently known as a science that studies changes in gene activity that take place without a change in the nucleotide sequence. Epigenetic modifications are transmitted from one cell generation to the next (mitotic inheritance) and can also be transmitted down organismal generations (meiotic inheritance) [55]. Epigenetic modifications may be induced by the incidence of environmental factors that affect these biological systems, making them important pathogenic mechanisms in complex multifactorial diseases. 

Epigenetic modifications include DNA methylation, histone modification, and those of the microRNA machinery. These mechanisms explain how cells with identical genomic information can differentiate into distinct cell types with different phenotypes [56]. Cytosine residues present in CG dinucleotides of gene promoters are targets for DNA methylation, which is associated with transcriptional silencing of these genes. This silencing can be caused by repressing transcription factor binding or by recruiting proteins that specifically bind to methylated CGs (methyl-CG-binding proteins, e.g., MeCP2), which can further recruit histone deacetyltransferases (HDACs) and corepressors. 

The enzymes that lead to DNA methylation are methyltransferases (DNMTs), of which there are types: DNMT1, which copies the pattern of DNA methylation during replication (methylation maintenance), and DNMT3a and DNMT3b, which are responsible for de novo DNA methylation [57]. In contrast, processes that demethylate DNA are not well understood. In eukaryotic cells genomic DNA is bundled with particular proteins, known as histones, to produce chromatin. The basic structure of chromatin is the nucleosome, which consists of 147 base pairs of DNA wrapped around an octamer of histone proteins consisting of an H3-H4 tetramer flanked on either side by an H2A-H2B dimer. Even if the core histones are densely packed, *histone modifying enzymes* can modify their NH2-terminal tails by acetylation, methylation, phosphorylation, sumoylation, or ubiquitination [58]. These modifications determine the accessibility of the DNA to the transcription machinery as well as being essential for replication, recombination and chromosomal organization. HDACs and histone acetyl transferases (HATs), respectively, remove and add acetyl groups to lysine residues on histone tails [59]. Although the levels of HAT activity and histone acetylation are known to correlate with those of gene transcription, the exact mechanisms promoting transcription are not very clear [60]. 

Native lysine residues on histone tails bear a positive charge that can bind negatively charged DNA to form a condensed structure with low transcriptional activity. An early suggestion was that histone acetylation removes these positive charges, thereby relaxing chromatin structure and facilitating access to the DNA for the transcriptional machinery to initiate transcription [60]. However, different models have recently been proposed wherein multiple histone modifications act in combination to regulate transcription [61]. Histone methylation can result in either transcriptional activation or inactivation, depending on the degree of methylation and the particular arginine and/or lysine residues modified. Histone methyltransferases and histone demethylases mediate these processes as well [62].

### 2.1. A possible Role of Epigenetic Mechanisms in GDM

As we previously commented, pregnancy is a pathological condition that may indicate a predisposition to develop T2D in near future. Moreover, several studies point towards GDM and T2D as different faces of the same disease at diverse stages of life [63]. High maternal adipose deposition or low levels of exercise contribute to insulin resistance in both pathologies [64–66] meanwhile b-cell dysfunction has also been recognized as an important factor to develop T2D and GDM [67]. In this case, sharing the same predisposing conditions and knowing that development of T2D is related to important epigenetic mechanisms [68], it may be consistent to identify the role of those epigenetic mechanisms in GDM pathogenesis.

In T2D, b-cell damage usually arises as a result of a combination of genetic susceptibility and acquired damage. Numerous genes have been associated with islet cell dysfunction in T2D, including some that encode for transcription factors, glucose metabolism proteins, and molecules of the insulin signaling pathways [69]. 

Pdx-1 is a pancreatic homeobox transcription factor that regulates pancreas development and b-cell differentiation. A reduction in Pdx-1 expression in humans and animal models has been shown to cause T2D, while mutations in this gene also cause the monogenic form of diabetes (MODY 4) [70]. Also, Park et al. have recently associated intrauterine growth retardation, impaired b-cell function, and T2D with the silencing of Pdx-1 by epigenetic mechanisms [71]. Previous works have related the increased risk of developing GDM with variants of MODY genes but further studies are necessary to establish the possible role of Pdx-1 in GDM [72].

Glucose is an important physiological fuel for the b-cell, initializing ATP production, insulin secretion, and proliferation. However, chronic hyperglycemia has severe adverse effects on b-cell function, including glucose desensitization, b-cell exhaustion and finally a lack of insulin secretion and insulin storage [73].

Increased oxidative stress found in obese and T2D patients, lead to decreased insulin gene transcription by decreasing Pdx1 [74]. The mechanisms by which ROS reduces b-cell mass and function are not completely understood, but ROS can alter DNA methylation. The changes in DNA methylation patterns have been shown to affect the expression of multiple genes [75, 76]. Histones, because they have abundant lysine residues, are also very susceptible to oxidative stress [77]. Several studies have identified epigenetic modifications in genes involved in the development of T2D, as we previously described for the Pdx-1 gene.

Glucose transporters are involved in the transport of glucose in most cells. GLUT4 is a member of the facilitative glucose transporter (GLUT) family, characterized by preferential expression in muscle and adipose tissues, where it is responsible for insulin-stimulated glucose uptake. GLUT4 is essential for the maintenance of normal glucose homeostasis [78]. 

In patients with T2D, insulin resistance is a product of decreased insulin-stimulated skeletal muscle glycogen synthesis, which can mostly be attributed to decreased insulin-stimulated glucose transport (Glut 4) activity [79] and similar alterations have been observed in maternal skeletal muscle in GDM [80, 81]. Glut 4 expression is further regulated by the interaction of the transcription factor MEF2 (myocyte enhancer factor 2) with HDAC5 (histone deacetylase 5), which deacetylates histone tails at Glut 4, resulting in a condensed chromatin structure and subsequently reduced Glut 4 expression [82]. Moreover, the MEF2 isoforms are differentially regulated in muscle and adipose tissue during periods of insulin deficiency [83]. These mechanisms could also be involved in Glut 4 alteration in GDM.

Insulin secretion in pancreatic islets is dependent upon mitochondrial function and production of ATP. The transcriptional coactivator peroxisome proliferator-activated receptor gamma coactivator-1 alpha (protein PGC-1alpha; gene PPARGC1A) is a master regulator of mitochondrial genes, and its expression is weak and related to impaired oxidative phosphorylation in the muscle of patients with T2D [84]. DNA methylation of the PPARGC1A promoter is greater in the pancreatic islets of patients with T2D than in those of healthy controls, which correlates inversely with PPARGC1A expression in diabetic islets. This weaker **PPARGC1A** expression is correlated with impaired glucose-stimulated insulin secretion, which is known to require ATP, and suggests that epigenetic mechanisms may regulate insulin secretion in human islets.

PPARGC1A is a coactivator of PPARG and PPARA and regulates genes involved in energy metabolism [85]. An alteration in peroxisome proliferator-activated receptors (PPARs) has been described in common complications of pregnancy, including GDM, intrauterine growth restriction (IUGR), and preeclampsia (PE) [86–88], which suggests the existence of a similar epigenetic control of the PPAR or PPARGC1A genes, as has been described for T2D.

### 2.2. Epigenetic Effects of GDM in Offspring

The epigenome is most vulnerable to dysregulation during those phases of life associated with higher rates of change for tissue development and growth, like embryogenesis, fetal, and neonatal life and puberty [89, 90]. 

Classical theories of disease development assumed that genetic susceptibility and unhealthy adult lifestyle give rise to insulin resistance and T2D, but observational studies showed a link between intrauterine growth retardation and subsequent development of T2D [91]. More recent theories based on a model of the developmental origins of health and disease (DOHaD) proposed that part of the susceptibility to T2D originates during intrauterine life as a result of altered environmental fetal programming, further amplified by subsequent rapid childhood growth. Fetal undernutrition (sometimes manifested as low birth weight) and postnatal overnutrition can both increase the risk of future diabetes [92]. This is also observed in animal models of intrauterine growth retardation caused by uteroplacental insufficiency, which limits the supply of critical substrates, nutrients, and hormones to the fetus. This abnormal metabolic intrauterine environment may affect the development of the fetus in humans by inducing changes in gene expression by epigenetic mechanisms of susceptible cells, leading to the development of diabetes in adulthood.

In the case of GDM, glucose travels freely from the mother to the fetus, but maternal insulin does not. Thus, maternal GDM exposes the fetus to higher concentrations of glucose than normal, forcing the fetus to increase its own insulin production [93]. GDM has serious, long-term consequences for both child and mother, including a predisposition to obesity, metabolic syndrome, and diabetes in later life [94]. The transgenerational persistence of the insulin resistant phenotype may be related to nutritionally induced epigenetic changes that influence the expression of key genes. The observed heritability of some epigenetic signatures in animal and human models suggests that the epigenotype can be transmitted to the next generation [95].

An adverse intrauterine milieu affects the development of the fetus by modifying expression in both pluripotential cells and terminally differentiated, poorly replicated cells and is associated with epigenetically induced downregulation of key genes controlling b-cell development, differentiation, and function [96–99].

## 3. Conclusions

Gestational diabetes is a growing health concern, especially in certain predisposed populations. Although traditionally deemed not as dangerous for the developing fetus as pregestational diabetes, recent findings indicate that GDM may have serious long-term consequences for both child and mother. 

A conceptual developmental disease framework for GDM emerges from the findings reviewed here. Epigenetic mechanisms may control insulin resistance in mothers during pregnancy diagnosed with GDM but could also predispose offspring to developing T2D in later stages of life. An increased need for insulin by the fetus to deal with the high levels of glucose caused by GDM is an environmental circumstance that probably triggers epigenetic changes in that early stage of life, involving genes critical to pancreatic development and b-cell function, peripheral glucose uptake and insulin resistance. Understanding the role of developmental programming genes is crucial to our understanding of GDM and its consequences for the mother and child and it might stimulate the development of further epigenetic therapeutic agents as modern tools for treating this disease.
